# Efficacy and Safety of Antiviral Agents in Preventing Allograft Rejection Following CMV Prophylaxis in High-Risk Kidney Transplantation: A Systematic Review and Network Meta-Analysis of Randomized Controlled Trials

**DOI:** 10.3389/fcimb.2022.865735

**Published:** 2022-04-01

**Authors:** Narisa Ruenroengbun, Tunlanut Sapankaew, Kamolpat Chaiyakittisopon, Pakpoom Phoompoung, Thundon Ngamprasertchai

**Affiliations:** ^1^ Department of Pharmaceutics (Clinical Pharmacy), Faculty of Pharmacy, Slipakorn University, Nakornprathom, Thailand; ^2^ Department of Internal Medicine, Faculty of Medicine, Thammasat University, Pathum Thani, Thailand; ^3^ Department of Community Pharmacy and Administrations, Faculty of Pharmacy, Slipakorn University, Nakornprathom, Thailand; ^4^ Department of Medicine, Faculty of Medicine Siriraj Hospital, Mahidol University, Bangkok, Thailand; ^5^ Department of Clinical Tropical Medicine, Faculty of Tropical Medicine, Mahidol University, Bangkok, Thailand

**Keywords:** CMV prophylaxis, allograft rejection, biopsy-proven acute rejection (BPAR), kidney transplantation, valganciclovir, ganciclovir, valacyclovir, network meta-analysis

## Abstract

Many antiviral agents have been studied in clinical trials for allograft rejection prevention following cytomegalovirus (CMV) prophylaxis in high-risk kidney transplant patients. However, data on the most effective and safest treatment are lacking. We conducted a systematic review and network meta-analysis to rank CMV prophylaxis agents for allograft rejection prevention following CMV prophylaxis in high-risk kidney transplant patients according to their efficacy and safety. We conducted searches on the MEDLINE, Embase, SCOPUS, and CENTRAL databases, as well as the reference lists of selected studies up to December 2021, for published and peer-reviewed randomized controlled trials assessing the efficacy of CMV prophylaxis agents in high-risk kidney transplant patients. Thirteen studies were independently selected by three reviewers and included post-kidney transplant patients indicated for CMV prophylaxis who had been randomized to receive prophylactic antiviral agents or standard of care. The reviewers independently extracted data from the included studies, and direct and network meta-analyses were applied to assess the study outcomes. The probability of efficacy and safety was evaluated, and the drugs were assigned a numerical ranking. We evaluated the risk of bias using the Cochrane Risk of Bias 2.0 tool. The primary outcome was an incidence of biopsy-proven acute rejection, whereas the secondary outcome was a composite of major adverse drug reactions. Each outcome referred to the definition provided in the original studies. Valganciclovir, valacyclovir, and ganciclovir were identified to significantly decrease the incidence of biopsy-proven acute rejection with pooled risk differences (RDs) of −20.53% (95% confidence interval [CI] = −36.09% to −4.98%), −19.3% (95% CI = −32.7% to −5.93%), and −10.4% (95% CI = −19.7% to −0.12%), respectively. The overall major adverse drug reaction was 5.7% without a significant difference when compared with placebo. Valganciclovir had the best combined efficacy and safety among the examined antiviral agents and was the most effective and safest antiviral agent overall for allograft rejection prevention following CMV prophylaxis. Valacyclovir was the optimal alternative antiviral agent for patients who were unable to tolerate intravenous ganciclovir or access oral valganciclovir as financial problem. However, compliance and dose-related toxicities should be closely monitored.

## Introduction

Even though kidney transplantation (KT) has increased survival in end-stage renal disease (Abecassis et al., 2008), infectious complications following KT have resulted in suboptimal outcomes, thus contributing to morbidity and mortality (Noppakun et al., 2015). Cytomegalovirus (CMV) is the leading cause of infectious etiologies resulting in high mortality (Razonable and Humar, 2019). The CMV disease spectrum ranges from asymptomatic infection to symptomatic CMV syndrome or invasive CMV disease. CMV infection in KT not only has a direct impact on targeted organs but also indirectly increases the risk for allograft rejection and other opportunistic infections (Ramanan and Razonable, 2013). CMV infection also indirectly stimulates adhesion and induces transforming growth factor (TGF)-β production, which leads to fibrogenesis (Helantera et al., 2003). Therefore, patients with CMV infection have a higher risk of allograft failure and death (Watcharananan et al., 2012; Raval et al., 2021). In a Thai study, the prevalence of asymptomatic CMV infection and CMV disease among KT recipients were 5%–21% and 7%, respectively (Watcharananan et al., 2012; Chiasakul et al., 2015). Older recipients receiving organs from CMV-seropositive donors or induction with anti-thymocyte globulin or lymphocyte-depleting agents were associated with CMV infection. Nearly all Thai donors and recipients were CMV seropositive, which indicates a moderate risk of CMV infection (Watcharananan et al., 2012; Chiasakul et al., 2015).

Prevention strategies against CMV infection vary among transplant centers in Thailand. A recent nationwide survey showed that almost 80% of Thai physicians implemented prevention strategies, including preemptive approaches (48%) and prophylaxis (45%). Prophylaxis is preferred for high-risk patients, such as those with a high level of immunosuppression (Bruminhent et al., 2019).

Currently, several antiviral agents are indicated for CMV prophylaxis in high-risk patients following KT, namely, valganciclovir (VGC), ganciclovir (GC), valacyclovir (VAC), and acyclovir (AC). The 2009 Kidney Disease: Improving Global Outcomes (KDIGO) guidelines recommend oral GC or VGC for CMV prophylaxis (Kasiske et al., 2010). VAC and AC are prescribed for CMV prophylaxis in current clinical practice (Kacer et al., 2015; Stamps et al., 2021). However, some drugs are not easily accessible in low- to middle-income countries. Additionally, VGC is expensive and is thus seldom used in general hospitals. However, KT has been increasingly performed in general hospitals in Thailand.

To our knowledge, previous systematic reviews and meta-analyses on CMV prophylaxis following KT have focused on the direct effects of antiviral agents on CMV infections, including CMV viremia or invasive CMV disease (Chatani et al., 2019; Ruenroengbun et al., 2021). The proper dosage of antiviral agents or preventive strategies in CMV prophylaxis following KT have also been reported (Florescu et al., 2014; Xin et al., 2017; Hwang et al., 2018; Lee et al., 2021). However, studies on antiviral agent efficacy against allograft rejection, as well as the relative efficacy and safety, are scarce. Therefore, we conducted a systematic review and network meta-analysis to estimate and rank the comparative efficacy and a composite of major adverse drug reactions (ADRs) of currently available antiviral agents against allograft rejection following CMV prophylaxis in high-risk patients.

## Methods

The systematic review and network meta-analysis protocol were developed following the guidelines of the Preferred Reporting Items for Systematic Reviews and Meta-Analyses for Network Meta-Analysis extension or PRISMA-NMA. Intention-to-treat analysis was conducted for all quantitative syntheses. The review protocol was registered with PROSPERO (CRD42019145845).

### Search Strategies

We searched for potential studies on the MEDLINE *via* PubMed, Embase, SCOPUS, and The Cochrane Central Registry of Controlled Trials (CENTRAL) databases up to December 2021. Two investigators (N.R. and K.C.) developed search strategies that were approved by the team. The search terms and strategies for each database were generated based on the targeted population, types of antiviral agents, and outcomes. The full search strategies are available in the **Supplementary Table 1**.

### Selection of Studies

The identified studies were independently selected on the basis of the title and abstract by two independent reviewers (N.R. and K.C.). Disagreement was resolved by discussion with a third reviewer (T.S.). Titles and abstracts were screened, and the full text was reviewed when a decision could not be made after reading the abstracts.

### Inclusion Criteria

We included randomized controlled trials (RCTs) in any language when they met the following criteria:

1. Participants were adult kidney transplant recipients who received prophylactic antiviral agents for CMV infection as indicated in primary articles;
2. The studies compared any two of the following interventions: AC, GC, VC, or any placebo;
3. The primary outcome was an incidence of biopsy-proven acute rejection (BPAR), and the secondary outcome was a composite of major ADRs;
4. The full text could be retrieved, and data were available for extraction; and
5. The study was published in a peer-reviewed journal or clinical trial registry.

Studies were excluded if foscarnet was used as an antiviral agent, as it has been indicated in CMV resistance treatment or intravenous immunoglobulin-containing regimen. Additionally, studies that reported BPAR without preceding CMV infection were excluded. Ongoing studies or study protocols from which we could not extract the results were also excluded

### Data Extraction

At least two of the three reviewers (N.R., K.C, and T.S.) independently extracted data using a standardized extraction form. The extracted data included the general characteristics of the articles, population, intervention, and outcomes of interest for pooling. Discrepancies were resolved by discussion or by a fourth reviewer (T.N.). Missing data were obtained by contacting the corresponding authors email or other researchers’ network platforms such as ResearchGate, Academia or Loop.

### Outcomes of Interest

The primary outcome of interest was BPAR associated with CMV infection after KT. The secondary outcome was a composite of major ADRs, including neutropenia, thrombocytopenia, leukopenia, anemia, and hallucinations.

### Risk of Bias Assessment

At least two of the three reviewers (N.R., K.C, and T.S.) independently evaluated the risk of bias of each study using the Cochrane Risk of Bias 2.0 tool for RCTs. The Risk of Bias 2.0 tool evaluates five domains of bias: randomization process, deviations from intended interventions, missing outcome data, measurement of the outcome, and selection of the reported results. The risk of bias for each of the five domains and the overall risk of bias were described as low, intermediate, or high. Disagreement between two authors was resolved by consensus and discussion. Cohen’s kappa was used to determine the agreement for each domain and the overall risk of bias.

### Statistical Analysis

We reported our systematic review following the PRISMA-NMA guidelines. Direct meta-analysis of comparisons between prophylactic antiviral agents for CMV infection and placebo or comparators was conducted by pooling risk difference (RD) if at least three studies were involved. The Q test and *I^2^* statistics were used to assess heterogeneity. If heterogeneity was present or the degree of heterogeneity (*I^2^*) was greater than 25%, the RDs were estimated using a random-effects model. Otherwise, a fixed-effects model was applied. We assessed publication bias using funnel plots and the Egger test.

For indirect comparisons, network meta-analysis was applied to determine the treatment effects for all possible treatment groups. A two-stage network meta-analysis was conducted to estimate the RD of all treatments using information from comparisons with common comparators to increase the power of the test by integrating direct and indirect comparisons. The consistency assumption (i.e., agreement between estimates from direct and indirect evidence) was also applied. In the comparison, AC, GC, VAC, VGC, and placebo or comparators were coded as 1, 2, 3, 4, and 0, respectively. Inconsistency was calculated as the difference between the indirect and direct estimates for a common comparator. The surface under the cumulative ranking curve (SUCRA) was used to rank the regimens in terms of efficacy and safety. A clustered ranking plot was used to present the benefits of BPAR prevention and safety according to the SUCRA. Publication bias was depicted using a comparison-adjusted funnel plot.

All analyses were conducted using STATA^®^ version 16.0 (StataCorp LP, College Station, TX, USA). A two-sided *p*-value of <0.05 was considered statistically significant, except in the heterogeneity test, in which a *p*-value of <0.1 was used.

## Results

### Study Characteristics of Included Studies

**Figure 1:**
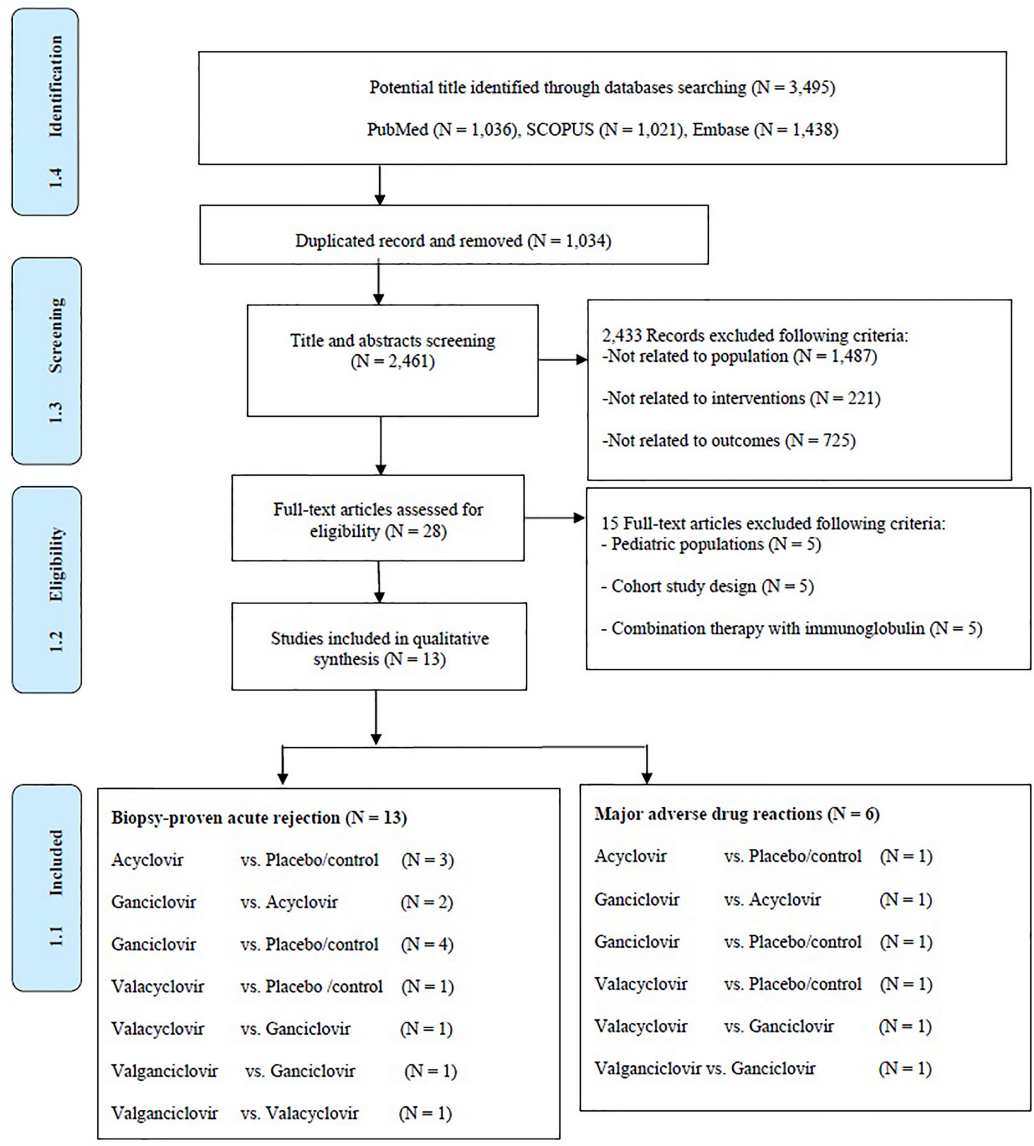
Preferred reporting items for systematic reviews and meta-analyses for network meta-analysis extension or PRISMA-NMA flow diagram of screening studies.

A total of 2,461 articles were identified, of which 2,433 were found ineligible and thus excluded (**Figure 1**). Overall, 13 RCTs were included for analysis. Five treatments were analyzed: AC, GC, VAC, VGC, and placebo. All these trials were conducted in developed countries, mostly in North America or Europe (**Table 1**). The trials recruited high-risk kidney transplant patients who were placed on a CMV prophylaxis regimen and compared antiviral prophylactic agents with placebo/comparators. Most of these studies were conducted during the 20^th^ century and mainly focused on GC, whereas oral antiviral agents were studied thereafter. The incidence of CMV infections reported, including CMV viremia or diseases following prophylactic antiviral agents, ranged from 11.7% to 90.6%. The incidence of CMV infections during the 20^th^ century was relatively high. Follow-up time among studies mostly ranged 6–12 months. Nearly all studies implemented lymphocyte-depleting antibody therapy. We assessed the included studies according to the outcome as follows: Thirteen studies reported BPAR, and six studies examined the rates composites of major ADRs.

**Table 1:** Characteristics of studies included in the review and network meta-analysis.

Study	N	CMV serostatusDonor and Recipient				Interventions	Comparator	BPAR		Major Adverse Drug Reactions		CMV Infection ^a^ n (%)	Follow-up time (months)	Lymphocyte-depleting antibody therapy^b^(%)	Maintenance immunosuppressive regimens^c^(%)			Countries
		D+/R+	D−/R+	D+/R−	D−/R−			Intervention (%)	Comparator (%)	Intervention (%)	Comparator (%)				Tacrolimus	Cyclosporine	Azathioprine	
Balfour et al. (1989)	104	31	43	30	0	Acyclovir 200–800 mg Q.I.D.	Control	15 (28.3)	12 (23.5)	1 (1.89)	3 (5.88)	57.7	3–12	8.65	NA			USA
Rondeau et al. (1993)	32	0	0	32	0	IV Ganciclovir 5 MKD B.I.D.	Control	10 (58.8)	9 (60.0)	NA	NA	75.0	3–12	18.8	NA			France
Rostaing et al. (1994)	37	17	20	0	0	Acyclovir 800 mg Q.I.D.	Control	7 (36.8)	8 (44.4)	NA	NA	64.9	3–12	100	–	70	30	France
Conti et al. (1995)	40	40		0	0	IV Ganciclovir 2.5 MKD	Control	8 (36.4)	13 (72.2)	0 (0.00)	0 (0.00)	35	12	95.0	NA			USA
Kletzmayr et al. (1996)	32	0	0	32	0	Acyclovir 200–800 mg Q.I.D.	Control	13 (59.1)	4 (40.0)	NA	NA	90.6	3–12	NA	–	100		Austria
Ahsan et al. (1998)	43	13	10	8	12	Ganciclovir 750 mg B.I.D.	Control	1 (4.5)	4 (18.2)	NA	NA	16.3	6	11.6	NA			USA
Brennan et al. (1997)	42	24	13	5	0	Ganciclovir 1,000 mg T.I.D.	Acyclovir	3 (20.0)	4 (19.0)	3 (20.0)	2 (9.52)	85.7	3–6	7.14	–	100		USA
Conti et al. (1997)	244	244		0	0	IV Ganciclovir 2.5 MKD B.I.D.	Control	31 (24.6)	58 (49.2)	NA	NA	22.9	12	NA	–	100		USA
Flechner et al. (1998)	101	29	23	27	0	Ganciclovir 1,000 mg T.I.D.	Acyclovir 800 mg Q.I.D.	7 (17.9)	13 (32.5)	NA	NA	13.9	3–6	100	–	100		USA
Lowance et al. (1999)	616	408		208		Valacyclovir 2,000 mg Q.I.D.	Control	107 (35.0)	179 (57.7)	37 (12.1)	36 (11.6)	32.6	3–6	16.6	–	81	–	USA and Europe
Paya et al. (2004)	120	0	0	120	0	Valganciclovir 900 mg O.D.	Ganciclovir 1,000 mg T.I.D.	17 (21.0)	9 (23.1)	NA	NA	11.7	6	NA	NA			USA
Reischig et al. (2005b)	83	60	13	10	0	Valacyclovir 2,000 mg Q.I.D.	Ganciclovir 1,000 mg T.I.D.	12 (33.3)	4 (11.4)	7 (19.4)	7 (20.0)	21.7	12	12.7	11	48	–	Czech
Reischig et al. (2018)	119	93	15	11	0	Valganciclovir 900 mg O.D.	Valacyclovir 2,000 mg Q.I.D.	8 (13.3)	15 (25.4)	21 (35.0)	13 (22.0)	35.0	6–36	50.4	49	51	–	Czech

D, donor; R, recipient; BPAR, biopsy-proven acute rejection; CMV, cytomegalovirus; KT, kidney transplantation; LT, liver transplantation; N, number of total patients in study; mg, milligram; O.D., once daily; B.I.D., twice a day; T.I.D., three times a day; Q.I.D., four times a day; IV, intravenous; MKD, milligram per kilogram per dose; m, month; y, year; NA, data not available.

a CMV infection included either CMV diseases or CMV viremia.

b Lymphocyte depleting antibody therapy included thymoglobulin, ATG, OKT-3, or alemtuzumab.

c All regimens contained mycophenolate mofetil and steroid.

### Risk of Bias Assessment Across the Included RCTs

The overall quality of the studies was rated as “some concerns” (**Supplementary Figure 1**). The highest quality was noted for the missing outcome data, whereas the lowest quality was noted for the randomization process domain.

### Direct Meta-Analysis

The incidence of BPAR was reduced by approximately 20% with GC (pooled RD = −0.22; 95% confidence interval [CI] = −0.32, −0.13; *p* < 0.01) compared with placebo (**Supplementary Figure 2**). Meanwhile, AC slightly increased the incidence of BPAR, but not significantly (pooled RD = 0.04; 95% CI = −0.09, 0.18; *p* = 0.53; **Supplementary Figure 3**). No publication bias was found, as assessed by the funnel plot for these outcomes (**Supplementary Figures 4, 5**). Less than three studies on ADRs were conducted on each agent; therefore, we were unable to conduct a direct meta-analysis.

### Network Meta-Analysis

**Figure 2:**
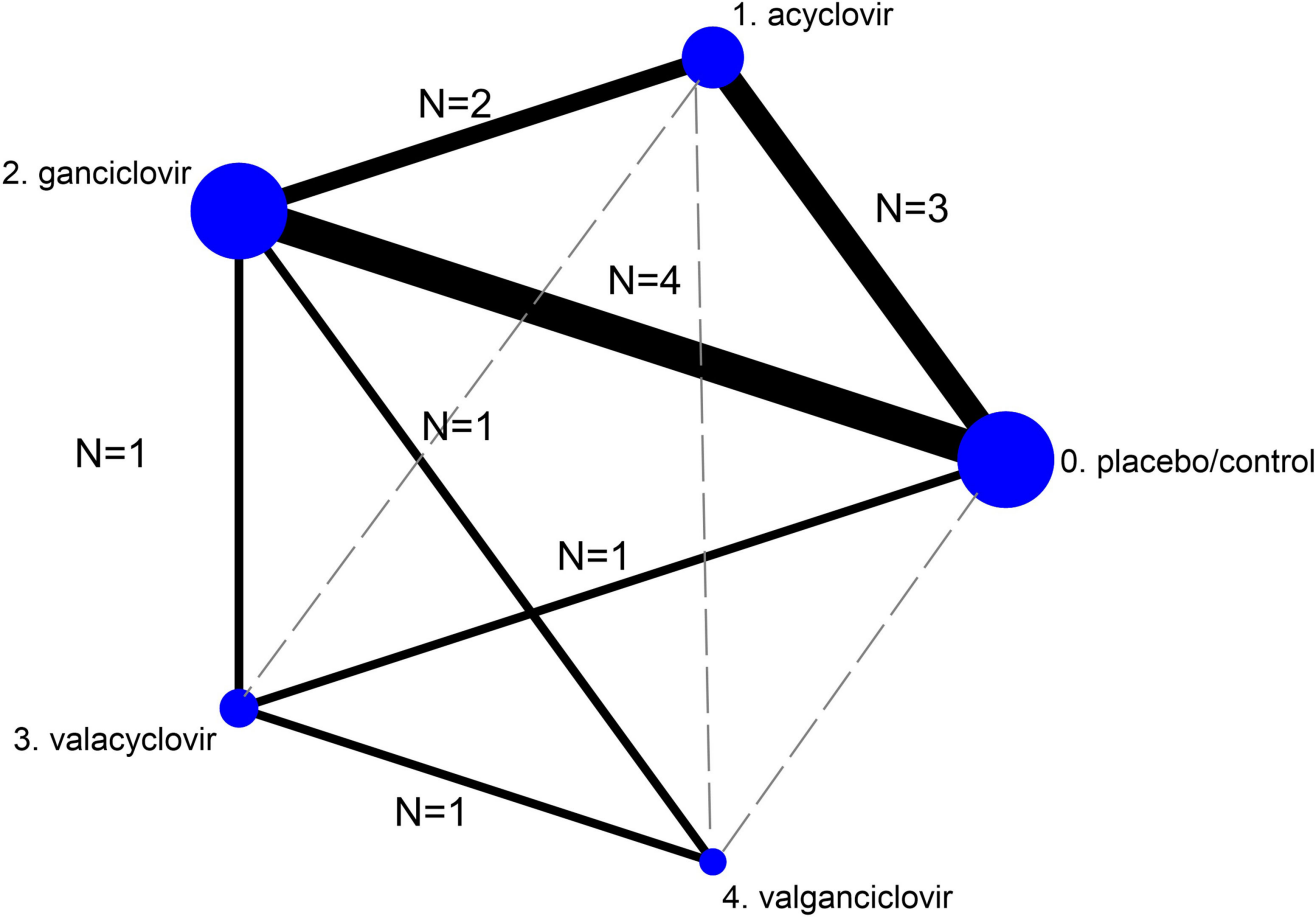
Network meta-analysis of eligible comparisons for efficacy (incidence of biopsy-proven acute rejection). The figure plots the network of direct comparisons (black bold lines) and indirect comparisons (dashed line). The width of the lines is proportional to the number of trials comparing every pair of treatments. The size of each circle is proportional to the number of randomly assigned participants (sample size).

**Figure 2** presents the network of eligible comparisons for BPAR incidence following CMV infection after KT. There was no evidence of inconsistency in efficacy and major adverse drug outcomes (chi-square = 7.52, *p* = 0.2621 and chi-square = 1.6, *p* = 0.6553, respectively). **Supplementary Figure 6** presents the network of eligible comparisons for the major adverse reactions.

### Incidence of BPAR

Incidence of BPAR from 13 studies (n = 1,613) consisting of seven direct comparisons among five treatments was pooled. Overall, VGC, VAC, and GC showed statistically better outcomes than did placebo. VGC particularly showed the highest risk reduction than did placebo with pooled RDs of −0.21 (95% CI = −0.36, −0.05), followed by VAC and GC with pooled RDs of −0.19 (95% CI = −0.33, −0.06) and −0.10 (95% CI = −0.20, −0.01), respectively (**Table 2**). The relative treatment efficacy among antiviral agents demonstrated that VGC most significantly reduced the incidence of BPAR, whereas AC slightly increased the risk of BPAR. VGC more significantly reduced the risk of BPAR than did VAC, GC, and AC with pooled RDs of −0.01 (95% CI = −0.15, 0.13), −0.10 (95% CI = −0.24, 0.04), and −0.12 (95% CI = −0.32, 0.08), respectively. VAC showed better BPAR reduction than did GC with pooled RDs of −0.09 (95% CI = −0.22, −0.04). The highest probability of efficacy regarding the incidence of BPAR, as indicated by the highest SUCRA, was identified for VGC, followed by VAC, GC, and AC.

### Safety Regarding a Composite of Major Adverse Drug Reactions

**Figure 3:**
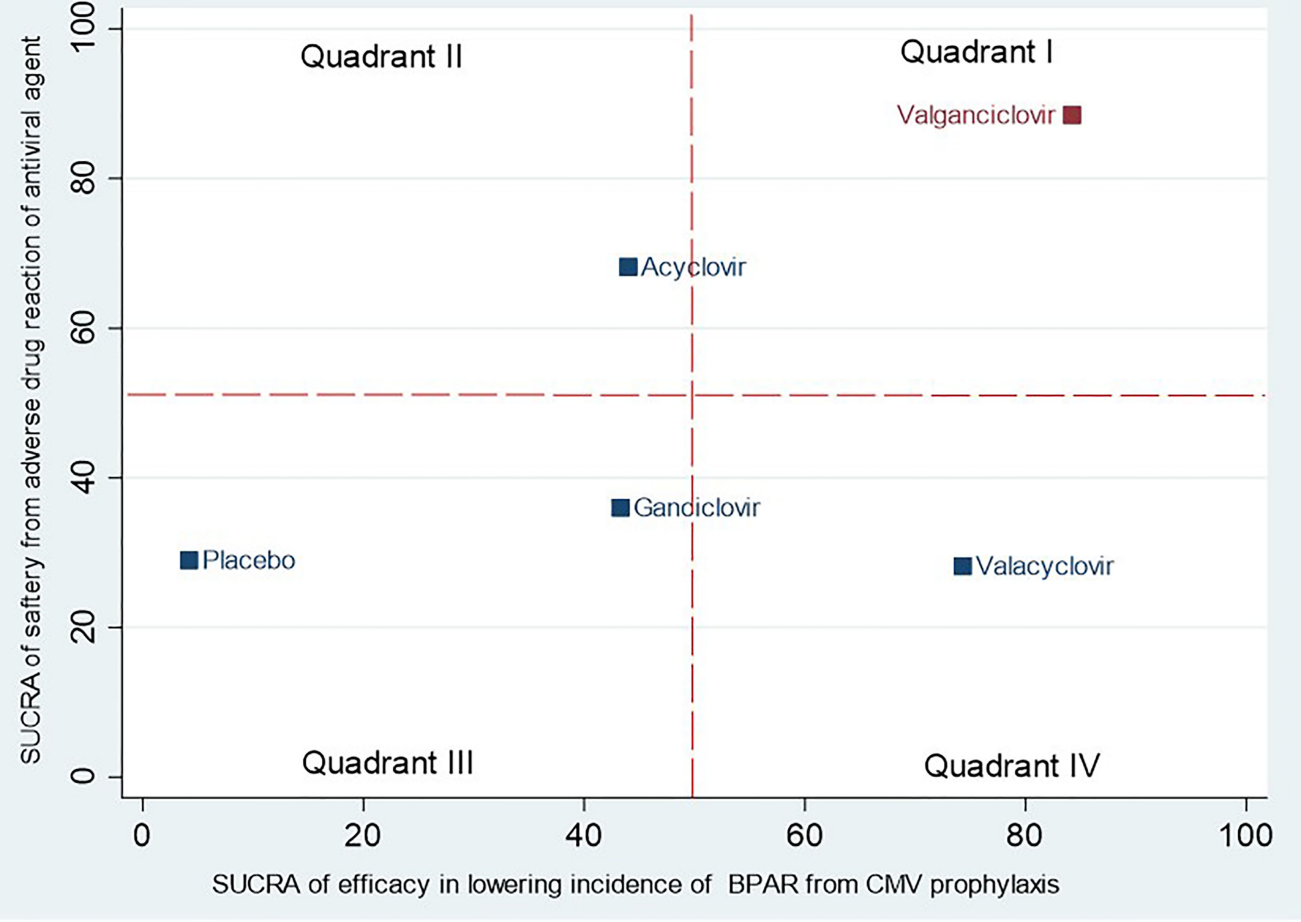
Cluster ranking plot of antiviral agents showing their surface under the cumulative ranking curve (SUCRA) values for the highest probability of incidence of biopsy-proven acute rejection (BPAR) versus SUCRA values for the lowest probability of major adverse drug reactions (ADRs). Each square point represents a group of antiviral agents in each cluster. Antiviral agents lying in the upper-right corner are associated with a higher probability of incidence of BPAR and a lower probability of major ADRs.

Overall, the composite of major ADRs was 5.7% without a significant difference when compared with placebo (**Table 2**). The relative major ADRs among the antiviral agents showed that VGC was the safest with approximately 10% fewer ADRs than comparators, whereas VAC slightly increased ADR compared with other comparators but without statistical significance (**Table 3**). The overall probabilities for efficacy and major ADRs were presented as cluster ranking plots (**Figure 3**). In terms of efficacy and low risk of ADRs, VGC was ranked highest for efficacy and safety and presented lying in the upper-right quadrant (SUCRA 84.2 and 88.5, respectively) (**Supplementary Table 2**). VAC was ranked second for efficacy (SUCRA 74.3) but had the highest risk for ADR (SUCRA 28.2) and indicated lying in the lower-right quadrant. Although AC and GC showed similar efficacies (SUCRA 44 and 47.3, accordingly), AC had fewer ADRs than GC.

**Table 2:** Pooled risk differences for the incidence of BPAR and major ADR among antiviral agents versus placebo/control.

Outcomes	Effect Sizes	Interventions			
		AC	GC	VAC	VGC
**BPAR**	RD (95% CI)	0.04 (−0.09, 0.18)	−0.10 (−0.20, −0.01)	−0.19 (−0.33, −0.06)	−0.21 (−0.36, −0.05)
	NNT (95% CI)	12 (5, NA)	10 (5, 476)	5 (3, 14)	5 (3, 75)
**Major ADR**	RD (95% CI)	−0.04 (−0.11, 0.03)	−0.003 (−0.079, 0.072)	−0.001 (−0.05, 0.05)	−0.11 (−0.29, 0.06)

BPAR, biopsy-proven graft rejection; ADR, adverse drug reaction; RD, risk differences; NNT, number needed to treat; AC, acyclovir; GC, ganciclovir; VAC, valacyclovir; VGC, valganciclovir.

**Table 3:** Results of the network meta-analysis of the incidence of BPAR and major ADR.

Interventions	Comparators	BPAR	MajorADR	Intervention	Comparators	BPAR	MajorADR
***AC***	PC	0.04 (−0.09, 0.18)	−0.04 (−0.11, 0.03)	**VAC**	PC	−0.19 (−0.33, −0.06)	0.00 (−0.05, 0.05)
	GC	0.02 (−0.13, 0.16)	−0.04 (−0.14, 0.06)		AC	−0.11 (−0.29, 0.08)	0.04 (−0.04, 0.13)
	VAC	0.11 (−0.08, 0.29)	−0.04 (−0.13, 0.04)		GC	−0.09 (−0.22, −0.04)	0.01 (−0.07, 0.09)
	VGC	0.12 (−0.08, 0.32)	0.07 (−0.11, 0.25)		VGC	0.01 (−0.13, 0.15)	0.11 (−0.06, 0.29)
***GC***	PC	−0.10 (−0.20, −0.01)	−0.01 (−0.08, 0.07)	**VGC**	PC	−0.21 (−0.36, −0.05)	−0.11 (−0.29, 0.06)
	AC	−0.02 (−0.16, 0.13)	0.04 (−0.06, 0.13)		AC	−0.12 (−0.32, 0.08)	−0.07 (−0.25, 0.11)
	VAC	0.09 (−0.04, 0.22)	−0.00 (−0.09, 0.08)		GC	−0.10 (−0.24, 0.04)	−0.11 (−0.27, 0.04)
	VGC	0.10 (−0.04, 0.24)	0.11 (−0.04, 0.27)		VAC	−0.01 (−0.15, 0.13)	−0.11 (−0.29, 0.06)

BPAR, biopsy-proven graft rejection; ADR, adverse drug reaction; NA, not applicable; AC, acyclovir; GC, ganciclovir; VAC, valacyclovir; VGC, valganciclovir.

### Publication Bias of the Network

Comparison-adjusted funnel plots were applied for the incidence of BPAR. No evidence of publication bias was found based on the symmetry of the funnel plot (**Supplementary Figure 7**).

## Discussion

Our systematic review revealed that antiviral agents contributed to the incidence of BPAR reduction which supported the benefit of antiviral agents to reduce the indirect effect of CMV on allograft failure (Watcharananan et al., 2012; Raval et al., 2021). Compared with placebo, GC reduced the incidence of BPAR following CMV prophylaxis in high-risk patients. Minimal increased incidence of BPAR was observed with AC. This is because AC confers no benefits on CMV infection and disease. This finding was consistent with previous studies (Rubin et al., 2000; Hodson et al., 2008; Ruenroengbun et al., 2021).

VGC showed the best comparative efficacy for allograft rejection prevention after CMV prophylaxis, followed by VAC and GC. VGC also had a lower pill burden than VAC and GC. Since high-risk recipients must undergo a CMV prophylaxis regimen for at least 3 months, compliance with the regimen should be considered. The pharmacokinetics of antiviral agents is another factor determining the optimal outcome. Given the high bioavailability of VGC, it demonstrated a 10-fold higher bioavailability than oral GC (Lake, 2003) and was as equally effective as oral GC in preventing CMV infection in solid-organ transplantation (Paya et al., 2004). It is also recommended in clinical practice guidelines (Kasiske et al., 2010). Similarly, VAC has a threefold to fivefold higher oral bioavailability than oral AC (Soul-Lawton et al., 1995). Several reports found that VAC was as effective as oral GC following KT (Reischig et al., 2002; Pavlopoulou et al., 2005; Reischig et al., 2005a). Most of the indicated Thai patients receive intravenous (IV) GC as a result of poor bioavailability of oral GC and inaccessibility to oral VGC as financial problem. However, CMV prophylaxis requires a long treatment duration of antiviral agents. Therefore, some patients are unable to tolerate IV GC. Additionally, VGC is rarely used in general hospitals, despite the gradual increase in KTs performed in these hospitals.

Our findings suggest that VAC would be a feasible alternative antiviral treatment for CMV prophylaxis in high-risk KT recipients. Nevertheless, higher dosage (8 g/day) was used in the aforementioned studies. High pill burden and neurotoxicity might limit its used in clinical practice. Consequently, compliance and dose-related toxicities should be closely monitored. In addition, current guidelines are not convincing regarding using VAC. Physicians should avoid AC for CMV prophylaxis, as its antiviral activities are ineffective against CMV infection. Moreover, minimal increased risk of BPAR were observed in AC group.

When the composite of major drug reactions was considered, VGC was ranked first for safety, followed by AC, GC, and VAC. Our findings thus support VGC recommendations in current guidelines in terms of efficacy and safety. Even though AC is safe, physicians should avoid its use because of its poor efficacy. The safety of GC was similar to that of VAC. We recommend VAC over GC in the Thai clinical setting for the aforementioned reason or for patients who are unable to tolerate GCV.

Our study has several strengths. First, we ranked the efficacy of antiviral agents on allograft rejection outcome. As this outcome determines survival of KT recipients. Second, the results have high generalizability and are highly applicable in low- to middle-income countries where VGC is not widely available. Lastly, we included the variety of CMV serostatus among donors and recipients which were generalized to both low and high-risk populations. However, this study has some limitations. The included studies presented heterogeneity regarding dosage and route administration of antiviral agents. Additionally, Other types of allograft rejection outcome, such as antibody-mediated rejection, were limited since most included studies were published before 2000. Finally, further large-scale and well-conducted RCTs are needed.

Overall, VGC was the best antiviral agent identified in terms of both efficacy and safety for allograft rejection prevention following CMV prophylaxis. VAC was identified as an optimal alternative for patients who are unable to tolerate GC or when oral GC is unavailable.

## Data Availability Statement

The original contributions presented in the study are included in the article/**Supplementary Material**. Further inquiries can be directed to the corresponding author.

## Author Contributions

Substantially contributed to conception or design: NR and TN. Contributed to identified and extracted data: NR, TS, and KC. Contributed to analysis, or interpretation of data: NR, TS, and KC. Drafted the manuscript for important content: NR and TN. Critically revised the manuscript for important intellectual content: PP and TN. All authors contributed to the article and approved the submitted version.

## Funding

No sponsorship for this study. The publication fee was funded by Faculty of Tropical Medicine, Mahidol University and Mahidol University, Bangkok, Thailand.

## Conflict of Interest

The authors declare that the research was conducted in the absence of any commercial or financial relationships that could be construed as a potential conflict of interest.

## Publisher’s Note

All claims expressed in this article are solely those of the authors and do not necessarily represent those of their affiliated organizations, or those of the publisher, the editors and the reviewers. Any product that may be evaluated in this article, or claim that may be made by its manufacturer, is not guaranteed or endorsed by the publisher.
