# Supplementary material for: Efficacy and Safety of Antiviral Agents in Preventing Allograft Rejection Following CMV Prophylaxis in High-Risk Kidney Transplantation: A Systematic Review and Network Meta-Analysis of Randomized Controlled Trials
Source: Front Cell Infect Microbiol. 2022 Apr 1;12:865735. doi: 10.3389/fcimb.2022.865735 (PMC9010655; doi:10.3389/fcimb.2022.865735)
Supplement: Supplementary file 1 [file DataSheet_1.docx]

**Supplementary Material**

**Table of contents Page**

Supplementary Table 1: Strategies searching using PICO framework. 1

Supplementary Table 2: Results of treatment ranking from network meta-analysis 6

of incidence BPAR and major adverse drug reactions

**Figures Page**

Supplementary Figure 1: Cochrane Risk of Bias 2.0 Tool of included randomized controlled trials 7

Supplementary Figure 2: Forest plot the efficacy of ganciclovir on incidence BPAR 8

in direct meta-analysis

Supplementary Figure 3: Forest plot the efficacy of acyclovir on incidence BPAR in 8

direct meta-analysis

Supplementary Figure 4: Funnel plot the efficacy of acyclovir on incidence BPAR 9

Supplementary Figure 5: Funnel plot the efficacy of ganciclovir on incidence BPAR 10

Supplementary Figure 6: Network diagram of major adverse reactions 11

Supplementary Figure 7: Comparison-adjusted funnel plot of incidence BPAR reduction 12

**Supplementary Table 1. Strategies searching using PICO framework.1.1 Search strategies and results in MEDLINE**

| **Domain** | **Search number** | **Query** |
| --- | --- | --- |
| **Patients (P)** | | |
| P1  (Kidney transplant) | #1 | Search kidney transplantation[MeSH Terms] |
|  | #2 | Search "kidney transplantation"[Text Word] |
|  | #3 | Search kidney transplant*[Text Word] |
| P2  (Renal transplant) | #4 | Search renal transplantation[MeSH Terms] |
|  | #5 | Search "renal transplantation"[Text Word] |
|  | #6 | Search renal transplant*[Text Word] |
| P3  (prophylaxis) | #7 | Search prophylaxis[Text Word]  Search prophyla*[Text Word]  Search (prophyla*[Text Word]) OR prophylaxis[Text Word] |
| P1 or P2 or P3 | #8 | #1 OR #2 OR #3 OR #4 OR #5 OR #6 OR #7 |
| **Interventions (I) and Comparators (C)** | | |
| I 1  (Valganciclovir) | #14 | Search valganciclovir[MeSH Terms] |
|  | #15 | Search "valganciclovir"[Text Word] |
| I 2  (Valaciclovir) | #16 | Search Valaciclovir[MeSH Terms] |
|  | #17 | Search “valaciclovir”[Text Word] |
| I 3  (ganciclovir) | #18 | Search ganciclovir[MeSH Terms] |
|  | #19 | Search “ganciclovir”[Text Word] |
| I 4  (acyclovir or aciclovir) | #20 | Search acyclovir[MeSH Terms] |
|  | #21 | Search "acyclovir"[Text Word] |
|  | #22 | Search "aciclovir"[Text Word] |
| I 1 or I2 or I 3 or I4 | #23 | #14 OR #15 OR #16 OR #17 OR #18 OR #19 OR #20 OR #21 OR #22 |
| **Outcomes (O)** | | |
| O 1  (CMV infection)  O2  (Biopsy Proven Acute Rejection) | #24 | Search cytomegalovirus infection[MeSH Terms] AND "CMV"[Text Word] |
|  | #25 | Search "cytomegalovirus infection"[Text Word] AND "CMV infection"[Text Word] AND Search CMV disease [Text Word |
|  | #26 | Search cytomegalovirus*[Text Word] AND CMV*[Text Word] AND arch cytomegalovirus disease[Text Word] |
|  | #27 | Search Acute graft rejection[Text Word] |
|  | #28 | Search Biopsy proven acute rejection [Text Word] |
|  | #29 | Search “BPRA” [Text Word] |
|  | #30 | Search “rejection*” [Text Word] |
|  | #31 | Search “graft rejection*” [Text Word] |
| O1 or O2 | #32 | #24 OR #25 OR #26 OR #27 OR #28 OR #29 OR #30 OR #31 |
| **P & I & O** | **#33** | **#13 AND #23 AND#32** |

- 1. **Search strategies and results in SCOPUS**

| **Domain** | **Search number** | **Query** |
| --- | --- | --- |
| **P (Patients)** | | |
| P1  (Kidney transplant) | #1 | TITLE-ABS-KEY ( "kidney transplantation" ) |
|  | #2 | TITLE-ABS-KEY ( kidney AND transplant* ) |
| P2  (Renal transplant) | #3 | TITLE-ABS-KEY ( "renal transplantation" ) |
|  | #4 | TITLE-ABS-KEY ( renal AND transplant* ) |
| P3  (prophylaxis) | #5 | TITLE-ABS-KEY (prophylaxis) |
| P1 or P2 or P3 | #6 | #1 OR #2 OR #3 OR #4 OR #5 |
| **Interventions and Comparators (I & C)** | | |
| I1: valganciclovir | #11 | TITLE-ABS-KEY ( valganciclovir ) |
| I2: valaciclovir | #12 | TITLE-ABS-KEY ( valaciclovir ) |
| I3: ganciclovir | #13 | TITLE-ABS-KEY ( ganciclovir ) |
| I4: acyclovir or aciclovir | #14 | TITLE-ABS-KEY ( acyclovir ) |
|  | #15 | TITLE-ABS-KEY ( aciclovir ) |
| I1 or I2 or I3 or I4 | #16 | #11 OR #12 OR #13 OR #14 OR #15 OR #16 |
| **Outcomes (O)** | | |
| O1  (CMV infection) | #17 | TITLE-ABS-KEY ( "cytomegalovirus infection" ) |
|  | #18 | TITLE-ABS-KEY ( cytomegalovirus* ) |
|  | #19 | TITLE-ABS-KEY ( "CMV" ) |
|  | #20 | TITLE-ABS-KEY ( "CMV infection" ) |
|  | #21 | TITLE-ABS-KEY ( cmv* ) |
| O2  Biopsy Proven Acute Rejection | #22 | TITLE-ABS-KEY ( biopsy proven acute rejection" ) AND TITLE-ABS-KEY (“BPAR”) AND “Graft rejection” AND TITLE-ABS-KEY (“rejection”) |
| O1 or O2 | #23 | #17 OR #18 OR #19 #20 OR #21 OR #22 |
| **P & I & C & O** | **#24** | **#10 AND #16 AND #23** |

- 1. **Search strategies and results in Embase**

| **Domain** | **Search number** | **Query** |
| --- | --- | --- |
| **Patients (P)** | | |
| P1  (Kidney transplant) | #1 | Search kidney transplantation[Emtree term] |
|  | #2 | Search 'kidney transplantation':ti,ab,kw |
|  | #3 | Search 'kidney transplant*':ti,ab,kw |
| P2  (Renal transplant) | #4 | Search renal transplantation [Emtree term] |
|  | #5 | Search 'renal transplantation':ti,ab,kw |
|  | #6 | Search 'renal transplant*':ti,ab,kw |
| P3  (prophylaxis) | #7 | Search prophylaxis *:ti,ab,kw |
| P1 or P2 or P3 | #8 | #1 OR #2 OR #3 OR #4 OR #5 OR #6 OR #7 |
| **Interventions and Comparators (I & C)** | | |
| I 1  (Valganciclovir) | #14 | Search 'valganciclovir'/exp[Emtree term] |
|  | #15 | Search "valganciclovir:ti,ab,kw |
| I 2  (Valaciclovir) | #16 | Search 'valaciclovir'/de[Emtree term] |
|  | #17 | Search valaciclovir:ti,ab,kw |
| I 3  (ganciclovir) | #18 | Search 'ganciclovir'/exp [Emtree term] |
|  | #19 | Search ganciclovir:ti,ab,kw |
| I 4  (acyclovir or aciclovir) | #20 | Search 'acyclovir'/exp[Emtree term] |
|  | #21 | Search 'acyclovir':ti,ab,kw |
|  | #22 | Search 'aciclovir':ti,ab,kw |
| I 1 or I2 or I 3 or I4 | #23 | #14 OR #15 OR #16 OR #17 OR #18 OR #19 OR #20 OR #21 OR #22 |
| **Outcomes (O)** | | |
| O 1  (CMV infection) | #24 | Search 'cytomegalovirus infection'/exp[Emtree term] |
|  | #25 | Search 'cytomegalovirus infection':ti,ab,kw |
|  | #26 | Search cytomegalovirus*:ti,ab,kw |
|  | #27 | Search 'cmv':ti,ab,kw |
|  | #28 | Search 'cmv infection':ti,ab,kw |
|  | #29 | Search 'cytomegalovirus disease':ti,ab,kw |
| O 2  Biopsy Proven Acute Rejection | #30 | Search ' biopsy proven acute rejection" AND “BPAR” AND “Graft rejection” AND “rejection” ':ti,ab,kw |
|  | #31 | Search “rejection” ':ti,ab,kw |
| O1 or O2 | #32 | #24 OR #25 OR #26 OR #27 OR #28 OR #29 OR #30 OR #31 |
| **P & I & C & O** | **#33** | **#13 AND #23 #32** |

| **Table 1.4 Gray literature searching** | |
| --- | --- |
|  |  |
| **Resources** | **Key words** |
| 1. Google scholar 2. OpenGrey sera 3. Mahidol university Library biomedical librarians 4. Gray Source Index 5. OpenDOAR directory of academicrepositories | - “Cytomegalovirus prophylaxis and biopsy proven acute rejection” - “Cytomegalovirus prophylaxis and BPRA” - “Cytomegalovirus prophylaxis and acute rejection” - “Cytomegalovirus prophylaxis and graft rejection” - “anti-CMV prophylaxis and reduce graft rejection” - “anti-CMV prophylaxis and biopsy proven acute rejection” - “anti-CMV prophylaxis and graft rejection” - “anti-CMV prophylaxis and renal graft rejection” - “anti-CMV prophylaxis and kidney graft rejection” |
|  | |
|  | |

**Supplementary Table 2. Results of treatment ranking from network meta-analysis of incidence BPAR and major adverse drug reactions**

| **Intervention** | **BPAR** | | **Major ADR** | |
| --- | --- | --- | --- | --- |
|  | **SUCRA** | **Pr** | **SUCRA** | **Pr** |
| PC | 12.2 | 0.0 | 29.0 | 0.0 |
| AC | 32.50 | 2.0 | 68.2 | 20.0 |
| GC | 46.3 | 16.0 | 36.0 | 1.0 |
| VAC | 74.8 | 31.0 | 28.2 | 2.0 |
| VGC | 84.2 | 58.0 | 88.5 | 77.0 |

BPAR= Biopsy-proven acute graft rejection, ADR = adverse drug reaction, PC=Placebo/Control, AC=Acyclovir, GC= Ganciclovir, VAC= Valacyclovir, VGC= Valacyclovir, Pr= probability of being the best treatment, SUCRA= surface under the cumulative ranking curve


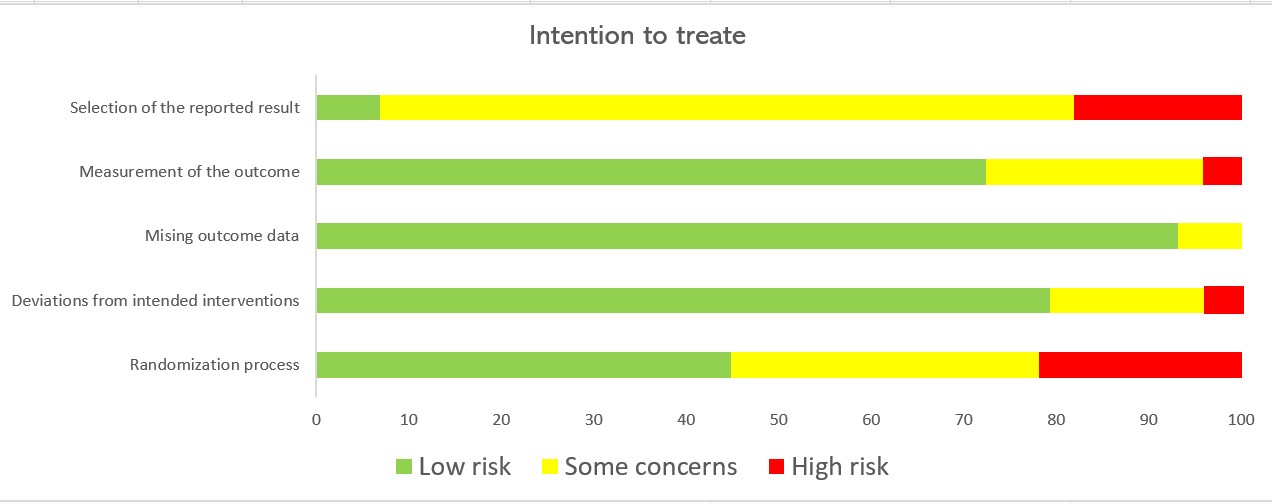


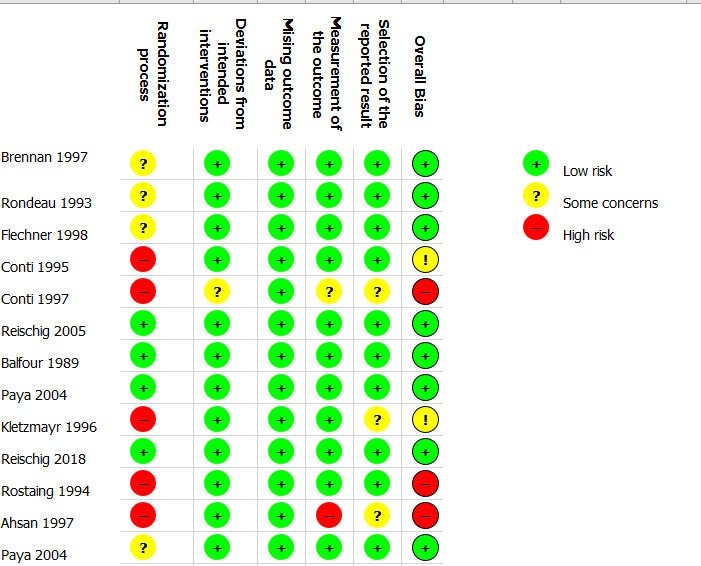


**Supplementary Figure 1: Cochrane Risk of Bias 2.0 Tool of included randomized controlled trials**

GC= Ganciclovir, PC= Placebo/Control

**Supplementary Figure 2. Forest plot the efficacy of ganciclovir on incidence BPAR in direct meta-analysis**

AC= Acyclovir, PC= Placebo/Control.

**Supplementary Figure 3. Forest plot the efficacy of acyclovir on incidence BPAR in direct meta-analysis**

**Supplementary Figure 4. Funnel plot the efficacy of acyclovir on incidence BPAR**

(egger’s test; *p* = 0.778)

**Supplementary Figure 5. Funnel plot the efficacy of ganciclovir on incidence**

(egger’s test; *p* = 0.896)

**Supplementary Figure 6: Network meta-analysis of eligible comparisons for major adverse reactions**

The figure plots the network of direct comparisons (black bold lines) and indirect comparisons (dashed line). The width of the lines is proportional to the number of trials comparing every pair of treatments. The size of each circle is proportional to the number of randomly assigned participants (sample size).

BPAR = Biopsy-proven acute rejection

**Supplementary Figure 7. Comparison-adjusted funnel plot of incidence BPAR**

A = Control, B = Acyclovir, C = Ganciclovir, D = Valacyclovir, E = Valganciclovir
